# Commentary: Analysis of head and neck cancer scRNA-seq data identified PRDM6 promotes tumor progression by modulating immune gene expression

**DOI:** 10.3389/fimmu.2025.1697007

**Published:** 2026-01-15

**Authors:** Min Li, Yingchun Zhou, Ming Cai

**Affiliations:** Department of Thyroid Oncology, Chongqing Key Laboratory of Translational Research for Cancer Metastasis and Individualized Treatment, Chongqing University Cancer Hospital, Chongqing, China

**Keywords:** head and neck cancer, PRDM6, immune genes, ScRNA-seq, HPV

## Introduction

The study by Wu et al., titled “Analysis of head and neck cancer scRNA-seq data identified PRDM6 promotes tumor progression by modulating immune gene expression,” published in Frontiers in Immunology, presents a compelling integration of single-cell RNA sequencing (scRNA-seq) and regulatory network analysis to uncover novel transcriptional mechanisms in head and neck squamous cell carcinoma (HNSCC) (1). By leveraging the IRIS3 platform to analyze multiple scRNA-seq datasets, the authors identify PRDM6 as a tumor-intrinsic epigenetic regulator that suppresses immune gene expression and promotes cell proliferation, particularly in the context of HPV infection. This work provides significant insights into the transcriptional control of immune evasion in HNSCC and highlights PRDM6 as a potential therapeutic target. However, several methodological and translational aspects warrant further critical evaluation. Furthermore, we extend the discussion to explore the translational potential of PRDM6 as a biomarker and therapeutic target, particularly in HPV-driven immune evasion.

## Subsections relevant for the subject

First, the use of IRIS3 for inferring cell-type-specific regulons represents a methodologically sound approach, leveraging both public ChIP-seq data and *de novo* motif prediction to enhance the robustness of TF–target identifications. However, IRIS3’s reliance on external ChIP-seq data—often derived from non-HNSCC cellular contexts—may introduce bias in regulon inference, as TF binding is highly context-dependent and influenced by cell type, tissue origin, and disease-specific chromatin states. This limitation may affect the accuracy of PRDM6 target gene predictions in HNSCC. Future studies should incorporate context-specific validation using HNSCC-derived ATAC-seq to map accessible chromatin regions, complemented by functional assays in patient-derived organoid models that better preserve tumor heterogeneity and native chromatin architecture (2, 3). Recent single-cell transcriptome studies have demonstrated the stepwise progression of HNSCC, highlighting the dynamic changes in tumor-immune interactions that could inform more precise regulon inference (4).

Second, the multi-omics validation—including scRNA-seq, bulk RNA-seq, ChIP-seq re-analysis, and functional assays in cell lines—strengthens the study’s conclusions. The demonstration that PRDM6 overexpression enhances proliferation and suppresses ISG expression is particularly convincing. However, the study did not explore whether PRDM6’s effects are mediated solely through H3K27me3 or involve other histone marks or non-histone targets. Additional epigenomic profiling would clarify the mechanistic basis of immune gene repression (5, 6). Notably, other PRDM family members, such as PRDM1, have been shown to modulate antitumor T cell activity (7), while the histone methyltransferase G9a, which interacts with PRDM family proteins, regulates chronic inflammation through non-canonical functions (8). These findings suggest that PRDM6 may exert immunosuppressive effects through both canonical and non-canonical pathways.

Third, the link between HPV oncoproteins E6/E7 and PRDM6 upregulation is a novel and clinically relevant finding. The consistent elevation of PRDM6 in HPV+ HNSCC samples from TCGA supports its role in viral-associated tumorigenesis. However, the exact mechanism by which E6/E7 transcriptionally activate PRDM6 remains unclear. Future work should investigate whether E6/E7 modulate PRDM6 through direct transcriptional activation, epigenetic remodeling, or via intermediate factors such as c-Myc or p53 (9, 10).

Fourth, while the study focuses on tumor-intrinsic immune regulation, the potential impact of PRDM6 on the tumor microenvironment (TME)—particularly on immune cell infiltration or function—was not addressed. Given that PRDM6 is expressed predominantly in tumor cells, its effect on T-cell exhaustion, macrophage polarization, or cytokine secretion would be of great interest for understanding systemic immune evasion (11, 12). Recent *in vivo* CRISPR screens have identified immune evasion pathways across cancers, highlighting how tumor-intrinsic factors like PRDM6 may limit type I interferon response and shape the immunosuppressive landscape (13).

We further hypothesize that PRDM6 may shape the immunosuppressive TME by altering cytokine secretion or checkpoint ligand expression. If PRDM6 regulates chemokine profiles or PD-L1 expression in HNSCC, its inhibition could potentially reinvigorate T-cell responses and synergize with anti-PD-1/PD-L1 therapies. Evaluating PRDM6’s extracellular role—such as via exosomal signaling—may uncover novel mechanisms of immune crosstalk and expand its biomarker utility.

Fifth, the exclusive use of cell line models (CAL27, SCC9) for functional validation, though practical, may not fully recapitulate the heterogeneity of primary HNSCC tumors. The inclusion of patient-derived organoids or xenograft models would enhance the translational relevance of the findings. Moreover, the lack of *in vivo* data limits the assessment of PRDM6’s role in tumor growth and immune evasion within a physiological context (14).

## Discussion

This study offers a robust, data-driven identification of PRDM6 as a key immune-regulatory TF in HNSCC, with strong implications for HPV-associated tumorigenesis. The integration of computational biology with experimental validation represents a notable strength. Looking forward, we propose concrete translational strategies to build upon these findings. First, combining PRDM6 inhibition with immune checkpoint blockade represents a promising therapeutic approach, particularly in HPV+ HNSCC where PRDM6-mediated immune suppression may contribute to immunotherapy resistance. Second, spatial transcriptomics could validate PRDM6’s role *in situ* by mapping its expression relative to immune cell infiltration and exclusion patterns within the tumor microenvironment. Third, developing PRDM6-specific small-molecule inhibitors or repurposing existing epigenetic drugs could provide tools to test whether reversing PRDM6-mediated repression sensitizes tumors to immunotherapy. Finally, PRDM6 expression could serve as a biomarker for patient stratification, identifying those most likely to benefit from combined epigenetic-immunotherapy approaches. These directions would accelerate the translation of PRDM6 biology into clinical applications for HNSCC treatment.

## References

[1] Wu Z Lepcha TT Zhou D He Z Fiches GN Park Y . Analysis of head and neck cancer scRNA-seq data identified PRDM6 promotes tumor progression by modulating immune gene expression. Front Immunol. (2025) 16:1596916. doi: 10.3389/fimmu.2025.1596916, PMID: 40936897 PMC12420620

[2] Ma A Wang C Chang Y Brennan FH McDermaid A Liu B . IRIS3: integrated cell-type-specific regulon inference server from single-cell RNA-Seq. Nucleic Acids Res. (2020) 48:W275–86. doi: 10.1093/nar/gkaa394, PMID: 32421805 PMC7319566

[3] ENCODE Project Consortium. An integrated encyclopedia of DNA elements in the human genome. Nature. (2012) 489:57–74. doi: 10.1038/nature11247, PMID: 22955616 PMC3439153

[4] Choi J-H Lee B-S Jang JY Lee YS Kim HJ Roh J . Single-cell transcriptome profiling of the stepwise progression of head and neck cancer. Nat Commun. (2023) 14:1055. doi: 10.1038/s41467-023-36691-x, PMID: 36828832 PMC9958029

[5] Schmidt C Cohen S Gudenas BL Husain S Carlson A Westelman S . PRDM6 promotes medulloblastoma by repressing chromatin accessibility and altering gene expression. Sci Rep. (2024) 14:16074. doi: 10.1038/s41598-024-66811-6, PMID: 38992221 PMC11239875

[6] Mzoughi S Tan YX Low D Guccione E . The role of PRDMs in cancer: one family, two sides. Curr Opin Genet Dev. (2016) 36:83–91. doi: 10.1016/j.gde.2016.03.009, PMID: 27153352

[7] Yoshikawa T Wu Z Inoue S Kasuya H Matsushita H Takahashi Y . Genetic ablation of PRDM1 in antitumor T cells enhances therapeutic efficacy of adoptive immunotherapy. Blood. (2022) 139:2156–72. doi: 10.1182/blood.2021012714, PMID: 34861037

[8] Muneer A Wang L Xie L Zhang F Wu B Mei L . Non-canonical function of histone methyltransferase G9a in the translational regulation of chronic inflammation. Cell Chem Biol. (2023) 30:1525–1541.e7. doi: 10.1016/j.chembiol.2023.09.012, PMID: 37858336 PMC11095832

[9] Peta E Sinigaglia A Masi G Di Camillo B Grassi A Trevisan M . HPV16 E6 and E7 upregulate the histone lysine demethylase KDM2B through the c-MYC/miR-146a-5p axys. Oncogene. (2018) 37:1654–68. doi: 10.1038/s41388-017-0083-1, PMID: 29335520

[10] Gewin L Myers H Kiyono T Galloway DA . Identification of a novel telomerase repressor that interacts with the human papillomavirus type-16 E6/E6-AP complex. Genes Dev. (2004) 18:2269–82. doi: 10.1101/gad.1214704, PMID: 15371341 PMC517520

[11] Wellenstein MD de Visser KE . Cancer-cell-intrinsic mechanisms shaping the tumor immune landscape. Immunity. (2018) 48:399–416. doi: 10.1016/j.immuni.2018.03.004, PMID: 29562192

[12] Chen YP Yin JH Li WF Li HJ Chen DP Zhang CJ . Single-cell transcriptomics reveals regulators underlying immune cell diversity and immune subtypes associated with prognosis in nasopharyngeal carcinoma. Cell Res. (2020) 30:1024–42. doi: 10.1038/s41422-020-0374-x, PMID: 32686767 PMC7784929

[13] Toufektchan E Dananberg A Striepen J Hickling JH Shim A Chen Y . Intratumoral TREX1 induction promotes immune evasion by limiting type I IFN. Cancer Immunol Res. (2024) 12:673–86. doi: 10.1158/2326-6066.CIR-23-1093, PMID: 38408184 PMC11148545

[14] Dickson MA Hahn WC Ino Y Ronfard V Wu JY Weinberg RA . Human keratinocytes that express hTERT and also bypass a p16(INK4a)-enforced mechanism that limits life span become immortal yet retain normal growth and differentiation characteristics. Mol Cell Biol. (2000) 20:1436–47. doi: 10.1128/MCB.20.4.1436-1447.2000, PMID: 10648628 PMC85304

